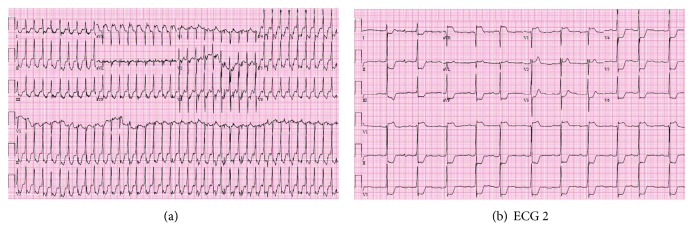# ST Segment Elevation and Depressions in Supraventricular Tachycardia without Coronary Artery Disease

**DOI:** 10.1155/2018/2716312

**Published:** 2018-12-13

**Authors:** Fuad Habash, Arwa Albashaireh, Mohammed Eid Madmani, Hakan Paydak

**Affiliations:** University of Arkansas for Medical Sciences, Little Rock, Arkansas, USA

## Abstract

ST segment changes are well documented in literature during supraventricular tachycardias. We present a case of a 21-year-old male who presents with chest pain, shortness of breath, and dizziness with an ECG showing atrioventricular reentrant tachycardia and diffuse ST segment depressions. Patient spontaneously converted to sinus rhythm, but he was still complaining of crushing chest pain. ECG taken after conversion showed sinus rhythm at a rate of 65 and showed obvious persistence of ST depressions in majority of leads. Emergent left heart catheterization showed normal coronaries. Such ST depression is suggestive of global ischemia in small intracardiac vessels that cannot be evaluated by left heart catheterization.

## 1. Introduction

Chest pain associated with ST changes is concerning for myocardial ischemia or infarction. In the settings of supraventricular tachycardia, ST depression can be seen but usually resolves after restoration of sinus rhythm. In this case report, we present a young patient who had supraventricular tachycardia with diffuse ST changes that remained after conversion to sinus rhythm.

## 2. Case Report

A 21-year-old man with history of uncontrolled hypertension and asthma presented to the emergency department (ED) with sudden onset substernal chest pain that started an hour before his arrival. Patient was walking down the stairs while at work and started having chest pain, sweating, and shortness of breath. Patient reported that he became dizzy and felt that his heart was racing. Although this episode of chest pain was unique and graded as severe, he previously had racing episodes that were not evaluated. No family history of cardiac disease was noted.

In ED, an ECG was obtained immediately at presentation (see Figure 1, ECG 1). ECG showed evidence of supraventricular tachycardia (SVT) at 220 beats per minute consistent with short RP tachycardia. ECG also showed diffuse ST segment depressions. Before any manoeuvres were applied, patient converted spontaneously to normal sinus rhythm but was still complaining of the same crushing chest pain.

A second ECG was obtained which showed significant diffuse ST depressions in leads I, II, III, AVF, V3, V4, V5, and V6 and ST segment elevation in leads AVR and V1 (see Figure 1, ECG 2). Although patient's rapid troponin test was negative, STEMI code pager was activated and the patient was transferred to cath lab emergently.

Heart catheterization showed normal coronary arteries. In addition, left ventricular ejection was estimated at 70% and his ascending aorta was normal without evidence of dissection. Troponin level hours later was positive and peaked at 10 ng·dL. On the next day, echocardiography was essentially normal with no wall motion abnormalities. Patient's electrolytes and thyroid function tests were within normal range. Patient was discharged on diltiazem. Later on, the patient underwent successful and uncomplicated slow pathway modification for the treatment of typical slow-fast AVNRT. No recurrence occurred at 6-month follow-up.

## 3. Discussion

This young man previously had palpitations but never sought medical help. This time, his palpitations were associated with severe chest pain that could not be ignored. His rhythm was fast with narrow QRS complexes, and ST segments in the first ECG are depressed in leads I, II, III, AVF, V3, V4, V5, and V6; ST segment elevation was also seen in leads AVR and V1. With such tachyarrhythmia, patient's heart could not compensate for its metabolic requirements reflecting as ECG changes of demand ischemia. Oddly, once he converted to normal sinus rhythm, his chest pain did not resolve and ST segment changes persisted even though the tachyarrhythmia was interrupted. These ST segment changes were very remarkable and required swift decision. Patient's coronaries were normal on coronary angiography. Cardiac walls were contracting normally, and no sequelae were seen on echocardiography to explain such electrical changes.

ST segment depression is well documented in literature during supraventricular tachycardias. These changes usually disappear after conversion to sinus rhythm, but it has been reported that ST depression can be still seen afterwards [1] [S8]. Slavich et al. suggest to observe such ECGs by the end of episodes [2]. There were no reports on ST segment elevation during AVNRT, and this is the first of its kind to our knowledge. The ST elevations in our patient's ECGs could not be missed, neither ignored, demanding emergent heart catheterization. We have no solid explanation to such ECG changes. As his epicardial coronary arteries did not show any pathology, we suggest that the patient had global ischemia in small intracardiac vessels that cannot be evaluated in cardiac angiography.

In one case series of 21 patients who presented with ST segment depression, 7 of them (33%) had significant coronary artery disease proven by angiography [3] [S7]. In the same series, they studied patients presenting with SVT but did not have any ST segment depression and all of the patients in this group had negative ischemic workup [3] [S7]. ST segment depressions could be indicative of coronary artery disease but is not the only mechanism for such ECG changes [3] [S07]. Another study showed that more than two-thirds of patient with SVT presenting with ST depression and troponin elevation have no coronary artery disease [4]. In addition, they did not find any correlation between the degree of ST segment depression and heart rate to the diagnosis of coronary artery disease. Also, Bukkapatnam et al. showed that ST segment depression and the increase of troponin were not significant predictors of CAD [4] [ref from S1 (3)].

Troponin elevation is expected when tachycardia ensues. About a third of patients with SVT have troponin elevation [5] [S5]. Numerous conditions other than myocardial infarction can be the cause; congestive heart disease, sepsis, pulmonary embolus, and thoracic injuries are most commonly seen [6] [S9]. The pathophysiology of such troponin elevations in non-ACS (non-acute coronary syndrome) is not well understood, but hypothesized to be due to endothelial dysfunction and demand ischemia or due to direct toxic effects of catecholamines [7] [S3]. In episodes of tachycardia, many researchers believe that the heart is craving more oxygen to fulfil its metabolic requirements but coronary blood flow happens during diastole which is shortened in tachycardia [1, 8, 9] [refs from S5 (13, 17, 19)]. Other authors think that myocardial stretch plays a role in troponin elevation through brain natriuretic peptide [8–10] [refs from S5 (17, 19, 20)]. Another hypothesis mentions increased permeability of myocardial cells during stress leading to troponin leak [11] [ref from S1 (7)].

Patients who had troponin elevation were found to have more comorbidities than those without [7] [S3]. In addition, patients with troponin elevation had increased risk of death, myocardial infarction, or rehospitalisation due to cardiac causes [7] [S3]. Other studies disagree and think that troponin elevation in SVT is not related to future outcomes [1, 12, 13] [refs from S3 (6, 30, 38)]. Controversies are probably due to different duration of follow-up of the studies and difference in demographics of the patients that were studied. For example, studying young patients with SVT and troponin elevation without comorbidities will have better outcome than patients who have multiple comorbidities and are old. Duration of tachycardia did not seem to correlate with the degree of troponin elevation [13] [S6].

A very recent study picked a random sample of patients with troponin elevations from hospital charts, 362/458 patients (79%) had troponin elevation that were contributed to non-ACS causes [6] [S9]. The first troponin elevations were 10, 0.4, and 0.14 in patients with STEMI, non-STEMI, and non-ACS, respectively [6] [S9]. Peak in STEMI was 34.7, peak in NSTEMI was 1.34, and peak in non-ACS was 0.21 [6] [S9]. Our patient had a troponin elevation similar to “STEMI” category but had normal coronaries.

### 3.1. Learning Points

The learning points of the study are as follows:

- Persistence of ST segment changes after restoration of sinus rhythm in patients presenting with SVT should be further evaluated for coronary artery disease
- Troponin leak in the setting of tachycardia is expected, yet further evaluation should be sought on individualised bases

## Figures and Tables

**Figure 1:**